# Early Complications After Percutaneous Closure of Atrial Septal Defect in Infants with Procedural Weight Less than 15 kg

**DOI:** 10.1007/s00246-016-1507-3

**Published:** 2016-11-11

**Authors:** Gustaf Tanghöj, Michal Odermarsky, Estelle Naumburg, Petru Liuba

**Affiliations:** 1 Department of Clinical Science Paediatrics, Umeå University, Umeå, Sweden; 2 Unit of Research Education and Development, Östersund Hospital, Östersund, Sweden; 3 Department of Cardiology, Pediatric Heart Center, Skåne University Hospital Lund, Lund University, Lund, Sweden; 4 Department of Paediatrics, Blekinge Hospital, Karlskrona, Sweden

**Keywords:** Atrial septal defect, Infant, Follow-up, Septal device, Post-interventional complications

## Abstract

Atrial septal defect (ASD) is the most common congenital cardiac lesion accounting for 10–15% of all cardiac malformations. In the majority of cases, the secundum type of the ASD is closed percutaneously in the catheterization laboratory. Although transcatheter closure of ASD is considered safe and effective in pediatric patients, there are limited data regarding the efficacy and safety of device ASD closure in smaller infants. The aim of this study was to determine risk of complications within 72 h following device closure of ASD in children of body weight <15 kg compared to larger children. Overall 252 children who underwent transcatheter closure of ASD at Children’s Heart Centre in Lund, Sweden, between 1998 and 2015 were included. Data regarding demographics, comorbidity and complications occurring during and after device procedure until discharge were retrieved from the hospital’s databases. Echocardiographic data were obtained from the digital and videotape recordings. Nearly half of the study cohort (*n* = 112; 44%) had a procedural weight <15 (median 11.3) kg with a median procedural age of 2.02 years. Among this study group, 22 (9%) children had post-procedural in-hospital complications, of which 16 (7%) were considered as major and six (2%) considered as minor. No deaths occurred. There was no significant difference in of the occurrence of major or minor complications between the two groups (*p* = 0.32). Larger ASD was more often associated with minor complications, OR 1.37 (95% CI 0.99–1.89), which most often consisted of transient arrhythmias during or after the procedure. Percutaneous ASD device closure can be performed safely in low-weight infants with a risk of post-procedural in-hospital complications comparable to larger/older children. Nevertheless, careful considerations of the indications to device closure is needed, particularly in children with larger ASD, as recommended by the current international guidelines for ASD closure.

## Background

Cardiovascular malformations affect approximately 800 children per year in Sweden [1, 2]. Atrial septal defect (ASD) is the most common congenital cardiac lesion accounting for 10–15% of all cardiovascular malformations [2].

Larger ASD causes a significant left-to-right shunt with subsequent volume load of the right side of the heart. If left untreated, particularly in children with comorbidities, changes in the pulmonary arterioles may lead to gradual rise in the pulmonary pressure and eventually irreversible arteriolar damage, which is known as Eisenmenger syndrome [3]. Surgery has been considered to be the standard treatment of ASD ever since the late 1960s with good long-term post-operative results [4, 5]. The first catheter-based closure of secundum ASD was described by King and Mills in 1976 [6]. Transcatheter closure of ASD has gradually become more common and has been associated with less complications and shorter hospitalization [7, 8]. Today, the transcatheter approach is considered safe and effective, with few but non-negligible short- and long-term adverse events [9].

Asymptomatic infants with a significant ASD shunt are typically followed until they have grown to an acceptable age and size. Recent guidelines recommend transcatheter closure after the age of 2, although common practice suggests a weight over 15 kg [10]. Symptomatic infants, such as those born prematurely, with failure to thrive or with additional comorbidity or need for respiratory support, may benefit from early ASD closure in order to improve their clinical status [11, 12]. Refinements in the device configuration and material used during catheterization have contributed to improved procedural safety even in small children with severe clinical picture [13, 14]. In older children and adults, increased ratio of the ASD diameter/weight ratio and deficient or absent aortic rim have been associated with increased frequency of adverse events following transcatheter ASD closure [15, 16]. Previous studies on the efficacy and safety in children with small body weight have focused on risk factors such as ASD size, procedural body weight and age [13, 14, 17, 18]. However, other factors such as cardiac and other comorbidity, genetic abnormalities, premature birth and pulmonary function including chronic lung disease (CLD) may influence the risk of in-hospital adverse complications following ASD device closure.

The aim of this study was to assess the risk of major and minor complications following percutaneous transcatheter device closure of ASD in children with procedural weight <15 kg compared to larger children. Other potential risk factors such as cardiac and other comorbidities including premature birth were also taken into account.

## Study Population and Methods

All children under the age of 18 years who underwent transcatheter ASD closure between 1998 and 2015 at the Skåne University Hospital in Lund, Sweden, were included in the study. Demographic information, such as age and weight at procedure, gestational age at birth and type of device, as well as information on complications and comorbidity was retrieved from medical records, from the Swedish Registry for Congenital Heart Disease (SWEDCON) and from the Swedish National Birth Registry (MFR) [19, 20]. MFR was established in 1973 and includes data on more than 99% of all births in Sweden [21]. Information on ASD size was retrieved from echocardiographic examinations. Comorbidity, including other cardiac defects, genetic abnormalities and other diseases, is retrieved from medical journals and classified according to Table 1. Gestational age was estimated by ultrasonic prenatal examination and retrieved from MFR register. Premature birth was defined as birth at <37 gestational weeks.

**Table 1:** Classification of comorbidity

Cardiac lesion	Genetic	Other
Pulmonary stenosis requiring treatment	Down syndrome and other trisomies	Cystic fibrosis
Pulmonary stenosis moderate/mild	Monosomies, translocations, deletions	Sickle cell anemia
Pulmonary stenosis, mild	Combinations of monosomy, deletions, trisomies	Omphalocele
Ventricular septal defect requiring treatment		Osler’s disease
Double outlet right ventricle		Muscular dystrophy
Transposition of great arteries		Diabetes type 1
Aortic coarctation		Bronchopulmonary dysplasia
Patent ductus arteriosus requiring treatment		Atrioventricular block
Pulmonary hypertension moderate to severe		Long QT syndrome
		Pulmonary hypertension, mild
		Bronchopulmonary Dysplasia
		Developmental delay

### Echocardiography

Echocardiographic data were retrieved from stored videotapes and digital examinations. The largest diameter of the ASD was measured from transesophageal echocardiography (TEE) images, being expressed in millimeter. All measurements were made by two investigators with intraobserver and interobserver variability of 3.0 and 2.7%, respectively.

The following indices were used:

1. The ASD diameter/weight ratio was calculated using the largest measured ASD diameter (from TEE) divided by the child’s weight at the procedure. The children were thereafter categorized in two groups using a cutoff ratio of 1.2. This value was earlier shown to be a risk factor for post-procedural complications [15, 18].
2. The ASD diameter/BSA ratio was calculated using the largest measured ASD diameter (from TTE) divided by the child’s BSA at the procedure. The body surface area (BSA) was calculated using Haycock geometrical formula [22].

Post-procedural complications were grouped into minor and major complications according to Bartakian et al. [17]. All complications occurring within the first 72 h after procedure or before discharge are classified and described in Table 2.

**Table 2:** Classification of complications

Major complications	Minor complications
Death	Deployment malfunction
Cardiac or/respiratory arrest	Suspected infection
Stroke	Bleeding not needing transfusion
Device erosion	Significant access site hematoma
Device embolization	Prolonged transient limb paresthesia
Permanent limb injury	Transient hypoxia during procedure
Need for emergent surgical procedure	Trivial pericardial/pleural effusion
Persistent therapy-requiring arrhythmias	
Events or intraprocedural arrhythmias requiring treatment	Transient arrhythmias not needing therapy (AV block, nodal rhythm, bradycardia)
Arrhythmias needing treatment	
Significant pericardial/pleural effusion	
Requiring treatment	
New valvular insufficiency/pulmonary vein obstruction	
Bleeding needing transfusion	
Pulmonary hypertension crisis	
Discharge later than 24-h post-closure	

### Statistical Analyses

Demographic data were analyzed using Student’s *t* test (unpaired two-sided) for parametrically distributed variables and Mann–Whitney U test for nonparametric distributed variables with a *p* value of <0.01 and Chi-squared test with a *p* value of <0.05 considered as statistically significant.

Demographic data are presented as mean and standard deviation (Std.) for parametric variables and as median and range for nonparametric variables.

Conditional logistic regression was preformed to evaluate the association between major and minor complication for children <15 kg at procedure and premature birth, cardiac and non-cardiac comorbidities, chromosomal abnormalities, ASD size, ASD diameter-to-weight ratio, BSA and ASD diameter-to-BSA ratio. Maximum likelihood estimates of the odds ratio (OR) and 95% confidence interval (CI) were obtained, taking into account potential confounding factors. The IBM SPSS Statistics, version 23 software (IBM Corporation, New York, USA), was used to fit the conditional logistic model.

The study was approved by the Ethics Committee for Human Research at the Lund University (D-nr 2015/559) in accordance with the ethical standards as laid down in the 1964 Declaration of Helsinki and its later amendments or comparable ethic standards.

## Results

In total, 252 children underwent ASD device closure at the Skåne University Hospital in Lund between 1998 and 2015. Their mean age was 5.36 (Std. 4.44) years, median weight was 15.60 (range 4.78–87.50) kg, and the mean ASD size was 12.15 mm (Std. 4.31 mm). The overall female-to-male ratio was 1.7:1. The demographic characteristics are shown in Table 3.

**Table 3:** Demographic characteristics of all children

	*n* (%)	Mean	Median	Std.	Min–Max
Weight (kg)	252	–	15.6	15.32	4.78–87.50
Age (years)	252	5.36	–	4.44	0.33–17.79
TEE ASD size (mm)	221	12.15	–	4.31	4.00–24.00
Gestational age	216	–	39	3.56	23.70–43.10
Device size	252	18.04	–	6.51	5.00–36.00
ASD/weight ratio (mm/kg)	221	0.70		0.42	0.13–2.53
ASD/BSA ratio (mm/m^2^)	221	18.33	–	9.12	3.20–53.34

Of these, 112 (44%) children had a procedural weight of <15 kg. Children ≤15 kg had a median weight of 11.30 (range 4.78–14.80) kg, whereas children >15 kg had a median weight of 23.50 (range 15.00–87.50) kg. Children ≤15 kg were younger with a mean age of 2.02 (Std. 1.00) years compared to children >15 kg with mean age of 8.03 (Std. 4.33) years (*p* < 0.00).

The overall comorbidity was present in 65 (26%) children. This included other cardiac defects in 22 (9%) children, genetic comorbidity in 25 (10%) and other in 31 (12%). Comorbidity was more frequent 42 (38%) in the group of children ≤15 kg compared to 23 (16%) in children >15 kg (*p* < 0.001). In total, 42 (17%) children were born preterm. Data on gestational week at birth were missing in 36 cases (14%), as these children were born abroad. Data on comorbidity are summarized in Table 4.

**Table 4:** Demographic information in each subgroup

	≤15 kg					>15 kg					
	*n (%)*	Mean	Median	Std.	Min–Max	*n (%)*	Mean	Median	Std.	Min–Max	*p*
Weight (kg)	112	–	11.30	2.48	4.78–14.80	140	–	23.50	16.19	15.00–87.50	<0.00
Age (years)	112	2.02	–	1.00	0.33–5.15	140	8.03	–	4.33	2.34–17.79	<0.00
TEE ASD size (mm)	96	10.97	–	3.40	5.00–21.00	125	13.05	–	4.72	4.00–24.00	<0.00
Gestational age	103	–	39.29	3.78	23.70–43.1	113	–	38.78	3.36	24.70–43.00	0.95
Device size	112	15.49	–	4.83	6.00–33.00	140	20.07	–	6.96	5.00–36.00	<0.00
ASD/weight ratio (mm/kg)	96	–	1.00	0.39	0.42–2.53	125	–	0.47	0.26	0.13–1.27	<0.00
ASD/BSA ratio (mm/m^2^)	96	24.76	–	8.61	9.73–53.34	125	13.39	–	5.86	3.20–30.29	<0.00
*Comorbidity*											* Chi 2*
Cardiac	13 (12)					9 (6)					0.15
Genetic	18 (16)					7 (5)					<0.00
Other	21 (19)					10 (7)					0.01
Preterm birth	24 (23)					18 (16)					0.17
*Device*											
Amplatzer	90					122					
Gore occluder	11					9					
Cardioseal	1					3					
Occlutech figulla flex	4					3					
Cocoon/vascular innovations	6					2					
Cardia atriasept	–					1					

Significance *p* < 0.05

The Amplatzer septal occluder was the most common type of device used (*n* = 212). Other devices included Gore Occluder (*n* = 20), Cocoon/Vascular Innovations (*n* = 8), Occlutech Figulla Flex (*n* = 7), Cardioseal (*n* = 4) and Cardia Atriasept (*n* = 1).

Major complications were more common than minor complications, especially in the group of children ≤15 kg. The type of complications in each group and in all children is shown in Table 5.

**Table 5:** Type of complications in each subgroup and in all children

	≤15 kg	>15 kg	All
*Numbers of patients*	112 (44%)	140 (56%)	252 (100%)
*Major complications*	11 (10%)	5 (4%)	16 (7%)
Need for emergent surgical procedure	*2 (2%)*	*2 (1%)*	*4 (2%)*
Arrhythmias requiring treatment	*1 (1%)*	*1 (1%)*	*2 (1%)*
Bleeding requiring transfusion	*2 (2%)*	*0*	*2 (1%)*
Pulmonary hypertension crisis	*1 (1%)*	*0*	*1 (1%)*
Blood pressure drop requiring treatment	*1 (1%)*	*0*	*1 (1%)*
Time to discharge >24 h	*4 (4%)*	*1 (1%)*	*5 (2%)*
Prolonged arrhythmias or potentially lethal events during procedure	0	1 (1%)	1 (1%)
*Minor complications*	1 (1%)	5 (4%)	6 (2%)
Arrhythmias not needing treatment		*4 (3%)*	*4 (12%)*
Bleeding not needing transfusion		*1 (1%)*	*1 (1%)*
Blood pressure drop	*1 (1%)*		*1 (1%)*

Significant *p* values are given in italic (*p* < 0.05)

### Echocardiography

ASD mean size by TEE was significantly smaller in children ≤15 kg 10.97 mm (Std 3.40 mm) compared to ASD size in children >15 kg 13.05 mm (Std. 4.72 mm) (*p* < 0.00). Also the device used was smaller 15.49 mm (Std. 4.83 mm) in children ≤15 kg compared to 20.07 mm (Std. 6.96 mm) in the other group (*p* < 0.000) (Table 4).

### Post-procedural Complications

Post-procedural in-hospital complications occurred in 22 (9%) children. There were no in-hospital deaths. Major complications occurred in 16 (7%) children, whereas minor complications occurred in six (2%) children, all on the first post-procedural day.

Major complications occurred in 11 (10%) children of ≤15 kg weight, compared to six (4%) children of >15 kg, with significant difference between the groups (*p* = 0.04). Minor complications occurred in one (1%) among children of ≤15 kg weight, compared to six (4%) among children of >15 kg, with no significant difference between the groups (Table 6).

**Table 6:** Risk factors for major and minor complications

Number of major complications (*n* = 16)	No	Yes	Total	*Chi-Square*
Weight ≤15 kg	101 (90%)	11 (10%)	112	0.04
Comorbidity	57 (88%)	8 (12%)	65	0.02
Cardiac comorbidity	19 (87%)	3 (14%)	21	0.14
Genetic comorbidity	21 (84%)	4 (16%)	25	0.04
Other comorbidity	27 (87%)	4 (13%)	31	0.11
Preterm	39 (93%)	3 (7%)	42	0.62
Number of minor complications (*N* = 6)				
Weight ≤15 kg	111 (99%)	1 (1%)	112	–
Comorbidity	64 (99%)	1 (%)	65	–
Cardiac comorbidity	22 (100%)	0	22	–
Genetic comorbidity	22 (100%)	0	22	–
Other comorbidity	31 (100%)	0	31	–
Preterm	42 (100%)	0	42	–

The majority of children (*n* = 242 (96%)) were discharged the day after the intervention. Six children were discharged within 1 week and 3 children within 2 weeks. One child was discharged 299 days after the ASD closure. Among children with late discharge, five suffered from post-procedural in-hospital complication (four major and one minor) and one child had fatal device erosion out of hospital 5 days after the procedure. In three of these children, there was no recorded post-procedural in-hospital complication. Down syndrome was present in 1 child, with a previous coarctation of the aorta, pulmonary hypertension and persistent ductus arteriosus, which delayed this patient from discharge. The child was hospitalized prior to the ASD closure due respiratory failure caused by a combination of congestive heart failure and pneumonia. By the time of intervention, the respiratory problems had resolved, and the delayed discharged was due to feeding support. One other child with chromosome 8 deletion, anal atresia, spherocytosis and respiratory failure needed continuous positive airway pressure and oxygen treatment 19 days prior to the ASD closure. After ASD closure, the respiratory support was continued and the child was extubated 26 h after the intervention. One child born preterm was discharged after 299 days of hospitalization. Prolonged hospitalization was mainly due to complex feeding, respiratory and neurological problems resulting in need of mechanical ventilation, tracheostomy and other care actions. One other child who was born preterm stayed more than 72 h in hospital following ASD device closure, due to severe respiratory problems related to tracheobronchomalacia. During this time, the child underwent tracheostomy and continuous mechanical ventilation. This child was also treated for multiple problems for months pre-intervention. Further, the child was treated for a suspected endocarditis, with a total hospital stay at several centers after the ASD closure of 299 days.

There was no significant association between any of the risk factors and major complication following ASD device closure. Neither was low weight, OR = 1.67 (95% CI 0.38–7.30), ASD diameter/weight ratio, OR = 0.92 (95% CI 0.13–6.63), nor ASD diameter/BSA ratio, OR = 1.05 (95% CI 0.96–1.16) associated with an increased risk of major complications in multivariate analysis (Table 7). An increased risk of minor complications was close to significant associated with a larger ASD size, OR = 1.37 (95% CI 0.99–1.89), after adjustments for confounding factors (Table 7).

**Table 7:** Risk of complications following ASD device closure, conditional logistic regression analyses

Univariate	Minor			Major		
	OR	*p* value	95% Cl	OR	*p* value	95% Cl
Weight ≤15 kg	0.24	0.20	0.03–2.31	2.94	0.05	0.99–8.73
ASD/BSA	0.93	0.34	0.81–1.08	1.08	<0.00	1.03–1.13
BSA	6.31	0.01	1.52–25.33	0.37	0.37	0.08–1.63
ASD/weight >1.2	0	–	–	4.18	0.02	1.31–13-35
Gestational age	0.99	0.94	0.76–1.29	0.96	0.60	0.83–1.11
ASD size	1.14	0.01	1.09–1.90	0.98	0.69	0.86–1.11
Device size	1.13	0.03	1.1–1.27	0.99	0.70	0.90–1.07
Card comorb	0	–	–	2.34	0.16	0.69–10.01
Gen comorb	1.85	0.58	0.20–16.50	3.41	0.05	1.01–11.53
Other comorb	0	–	–	2.57	0.12	0.77–8.53
*Multivariate*						
Weight ≤15 kg	0	–	–	1.67	0.50	0.38–7.30
ASD size	1.37	0.05	0.99–1.89	–		
BSA	1.64	0.72	0.12–23.39	–		
Device size	0.96	0.72	0.78–1.19	–		
ASD/BSA	–			1.05	0.30	0.96–1.16
ASD/weight >1.2	–			0.92	0.93	0.13–6.63
Card comorb	–			2.13	0.32	0.48–9.50
Gen comorb	–			1.91	0.35	0.50–7.30
Other comorb	–			2.09	0.28	0.55–7.97

## Discussion

Our retrospective survey of a large cohort of children with device closure of the ASD indicates a relatively low rate of early post-procedural complications in those weighing <15 kg. The risk of complications in this cohort of patients was not altered by the presence of additional comorbidity or large ASD size.

We found an overall post-procedural in-hospital complication rate of 8.7%, with 9.8% in the group of children of ≤15 kg compared to 7.1% among children of >15 kg, but no significant difference between the groups. This is in line with previous studies on complications following percutaneous ASD closure [9, 17].

In children ≤15 kg at the procedure, major complications were more frequent (10%) compared to children >15 kg (4%) at the procedure. However, we included discharge from hospital later than 24 h as a major complication, which accounted for 5 cases in our study. These children suffered from severe pre-procedural medical conditions and needed longer post-procedural care, unrelated to the device intervention. A complex medical history prior to ASD closure may thus lead to longer hospital stay and to several non-procedural-related complications such as feeding support and respiratory support. When excluding discharge later than 24 h as a major complication after ASD closure, the overall complication rate for the total study group dropped to 6.3% and the rate of major complications to 4.3%. This is in line with other studies [17]. Comorbidity was more common among children of ≤15 kg compared to children >15 kg. In four cases, a prolonged hospital stay post-intervention was associated with the compounded comorbidity. However, when recalculating complication frequencies without using prolonged hospital stay post-intervention as an event no significant difference in the frequencies of major complications was found.

Higher ASD diameter/weight ratio and ASD-to-device size ratio have been earlier associated with greater risk of peri-procedural complications [15, 18]. In our study, we adjusted risk estimations for minor as well as major complications for these and other potential risk factors. The ASD size and device size were larger among children >15 kg in our study. The ASD size-to-body weight ratio was doubled among the smaller children compared to those >15 kg. Neither ASD diameter/weight ratio nor ASD diameter/BSA ratio was associated with an increased risk of major or minor complications in our study. However, the risk of minor complications increased with larger ASD size, and this association remained near significant after adjustments for other risk factors OR = 1.37 (95% CI 0.99–1.89). No such risk was noticed for major complications. Increased frequency of minor complications, such as transient arrhythmias, following percutaneous closure of larger ASD (>15 mm) has been reported by others [23]. Altogether, four children in our study were identified with procedural-related arrhythmias, which did not need treatment.

A previous study concluded that device closure of ASD >20 mm in children <20 kg is safe with regard to the risk of major complications [24]. We performed subgroup analysis of the risk of in-hospital complication after closure of ASD >20 mm. Overall 14 (6%) children and only one (1%) child of ≤15 kg had an ASD diameter larger than 20 mm. The risk of post-procedural minor complications for these children was associated with OR = 44.87 (95% CI 2.29–877.95, *p* = 0.012) after adjustments for potential confounders. Even though this accounted for a small group of patients, the risk of minor complications indicated that careful intra- and post-procedural monitoring of these children is needed.

Most of the minor complications in our study were intraprocedural arrhythmias. Thus, our study supports the hypothesis that a combination of abnormally stretched atrial wall decreased myocardial mass and edema due to a large device may trigger transient arrhythmias [24, 25]. ASD is known to alter the atrial depolarization as well as the structure of the atria with increased risk of arrhythmias [26, 27]. Although closing the ASD may reduce the prevalence of atrial tachyarrhythmia [28], transient arrhythmias including supraventricular tachycardia, ectopy and atrioventricular block have been described following ASD closure.

Spontaneous closure of ASD <8 mm is known to occur during the first years of infancy, but some ASD may enlarge over time [29]. In our study, the cohort of children of ≤15 kg (ASD size 5–21 mm) might include some ASDs with a potential of spontaneous closure later in life. However, all children included in the study had right heart enlargement as sign of significant ASD shunt, so this possibility is less likely.

In our study, comorbidity was more common among the smaller children, which is in agreement with others [23]. Genetic abnormalities, including Down syndrome, was also more common among the smaller children in our study, 16% of children ≤15 kg compared to 5% of children >15 kg. Neither genetic nor cardiac comorbidity was associated with increased risk of complications. Down syndrome is known to increase risk of pulmonary hypertension as well as heart failure in the presence of shunt defects [30, 31]. The incidence of ASD among children with Down syndrome is 8–48% [32]. In our study, 15 (6%) of all children had Down syndrome.

Several earlier studies have showed that children suffering from Down syndrome with compound cardiovascular malformations may benefit from corrective surgery of ASD [31, 33, 34]. Our study indicates that percutaneous ASD closure can be preformed safe, but the patients with Down syndrome were few and larger studies are needed.

Cardiovascular malformations are twice as common among preterm infants compared to full-term infants and have an increased mortality rate [35, 36]. The overall incidence of ASD among prematurely born infants is unknown, but a delayed spontaneous closure of ASD has been noted [36]. In our study, 17% of all infants with ASD percutaneous closure were born preterm and occurred among the children ≤15 kg (23%) compared to those of >15 kg (16%), with nonsignificant difference between the groups. Larger studies are needed to determine the risk of ASD device closure among preterm born children.

Prematurely born infants may suffer from severe pulmonary complications in association with ASD and pulmonary hypertension even as adults [12, 37–39]. Thus, prematurely born children may benefit from early percutaneous treatment to eliminating the left-to-right shunt [12, 37–39]. Prematurity was not a risk factor in our study for neither minor nor major complications, indicating that prematurely born infants can be considered for percutaneous closure of ASD. Even if ASD closure is safe for prematurely born infants, longer follow-up studies are needed to determine the long-term safety and benefits of early ASD closure.

Our study is limited by its retrospective nature. Data are collected from medical journals, registers and echocardiographic examinations, and thus, there is a risk of selection bias and missing data. To reduce these risks, all echocardiographic data were retrieved by only two researches with very low intraobserver and interobserver variability. Further, the number of included children is substantial, contributing increased power.

Using already assigned entities of complications to either major or minor categories may strengthen the study in comparison with other. Lund is one of the three centers in Sweden where percutaneous treatment of ASD is performed. During the study period, only 3 interventionists preformed all ASD closures. This minimizes risk of selection bias due to different techniques and interventionist skills.

## Conclusion

ASD closure in most infants with a bodyweight of below than 15 kg or less can be performed safely with a low risk of post-procedural in-hospital complications, but considerations due to non-negligible complications can occur and sufficient indication to benefit the closure in small infants must be carefully considered. Increased risk of minor complications after ASD closure was associated with an increasing size of ASD. Further studies are needed to establish the long-term safety of ASD closure via device on low-weight children with potential risk factors such as prematurity, cardiac and other comorbidities.
